# Impact of dog and/or cat ownership on functional constipation at 3 years of age: the Japan Environment and Children’s study

**DOI:** 10.1186/s12887-023-04412-4

**Published:** 2023-11-23

**Authors:** Noriko Motoki, Yuji Inaba, Hirokazu Toubou, Kohei Hasegawa, Takumi Shibazaki, Teruomi Tsukahara, Tetsuo Nomiyama, the Japan Environment and Children’s Study (JECS) Group, Michihiro Kamijima, Shin Yamazaki, Yukihiro Ohya, Reiko Kishi, Nobuo Yaegashi, Koichi Hashimoto, Chisato Mori, Shuichi Ito, Zentaro Yamagata, Hidekuni Inadera, Takeo Nakayama, Tomotaka Sobue, Masayuki Shima, Hiroshige Nakamura, Narufumi Suganuma, Koichi Kusuhara, Takahiko Katoh

**Affiliations:** 1 Center for Perinatal, Pediatric, and Environmental Epidemiology, Shinshu University School of Medicine, Matsumoto, Nagano, Japan; 2 Department of Pediatrics, Shinshu University School of Medicine, Asahi 3-1-1, Matsumoto, Nagano, 390-8621 Japan; 3 Department of Neurology, Nagano Children’s Hospital, Azumino, Nagano, Japan; 4 Life Science Research Center, Nagano Children’s Hospital, Azumino, Nagano, Japan; 5 Department of Preventive Medicine and Public Health, Shinshu University School of Medicine, Matsumoto, Nagano, Japan

**Keywords:** Dog, Cat, Pet ownership, Functional constipation, Children

## Abstract

**Purpose:**

This investigation assessed the impact of dog and/or cat ownership during infancy on the presence of functional constipation (FC) at 3 years of age.

**Methods:**

The fixed data of 73,936 singleton births from a large national birth cohort study commencing in 2011 were used to identify FC as estimated by Rome III at 3 years of age. Multiple logistic regression analysis was employed to search for correlations between FC development and dog and/or cat ownership in early childhood.

**Results:**

A total of 8,459 toddlers (11.6%) met the Rome III criteria for FC at 3 years of age. Overall, 57,264 (77.5%) participants had never owned a dog or cat. We identified 7,715 (10.4%) infant-period owners, 1,295 (1.8%) current owners, and 7,762 (10.5%) long-term owners. Multivariate analysis showed that infant-period ownership remained significantly associated with the risk of developing FC at 3 years of age after adjusting for covariates (adjusted OR [95% CI] 1.09 [1.01–1.19] based on non-ownership).

**Conclusions:**

This Japanese large nationwide survey uncovered a possible adverse effect of infant-period dog and/or cat ownership prior to 6 months of age on FC status at 3 years of age.

**Supplementary Information:**

The online version contains supplementary material available at 10.1186/s12887-023-04412-4.

## Introduction

Functional constipation (FC) is characterized by chronic or recurrent constipation with no identifiable underlying cause. Although FC is a frequent condition in childhood, it can significantly impair the quality of life of the child and parents, leading to frequent and costly medical services [1]. FC in children is considered a heterogeneous disorder, with such multifactorial causes as dietary habits and lower intake of fruits and vegetables, less outdoor play activity, and lower regular physical activity time [2–5]. Family problems and psychological stress have also been associated with constipation during school age [5]. However, the etiology of FC has not been fully clarified.

The pet ownership rate is high in many developed countries, with approximately 38% of households owning at least 1 dog in the United States [6]. Contact with companion animals contributes to human health by reducing stress and improving mental health [7]. Moreover, pet ownership provides benefits for adults in terms of physical activity, social function, and cardiovascular risk [7, 8]. Previous studies have shown that attachment to a pet is related to physical, psychological, and social benefits in children [9–11]. Dog contact during infancy may have a protective effect on respiratory tract infection and otitis media [12]. Household dog or cat ownership is also associated with a reduced risk of gastroenteritis in young children, possibly through acquired immunity via low-level chronic exposure to potential microbial contaminants [13], with several reports on the effects of pet ownership on gut microbiota profiles [14–19]. On the other hand, an imbalance in intestinal microbiota is considered an etiological factor for FC in children [20]. There have been no studies to date on the relationship between domestic dog and/or cat ownership during infancy and FC development. Accordingly, we conducted a large birth cohort study to investigate the impact of dog and/or cat ownership during infancy on the presence of FC at 3 years of age.

## Methods

### Study design and participants

The data regarding pet exposure and FC were procured from the Japan Environment and Children’s Study (JECS), which is an ongoing cohort study that commenced in January 2011 to investigate the impact of environmental factors on children’s health.

In the JECS, study enrollment and survey information collection for pregnant women began in January 2011. Pregnant women were enrolled at among 15 Regional Centers (Hokkaido, Miyagi, Fukushima, Chiba, Kanagawa, Koshin, Toyama, Aichi, Kyoto, Osaka, Hyogo, Tottori, Kochi, Fukuoka, and South Kyushu/Okinawa) in Japan between January 2011 and March 2014. The inclusion criteria were: 1) having residence in the Study Area at the time of recruitment, 2) expected delivery after August 1, 2011, and 3) capable of comprehending the Japanese language and completing the self-administered structured questionnaire in Japanese. Survey information was obtained during the first and second/third trimester from the pregnant women. Detailed information regarding the mothers and their children was derived from medical record transcripts prepared by physicians, midwives/nurses, and/or Research Co-ordinators during the first trimester, at delivery, and when the child was 1 month old. After delivery, medical data were collected at the age of 1 month and then every 6 months via self-administered questionnaires by caregivers, still ongoing.

The JECS has been registered in the UMIN Clinical Trials Registry (no. UMIN000030786). Details of the JECS project were described previously [21–23]. The JECS protocol was reviewed and approved by the Ministry of the Environment’s Institutional Review Board on Epidemiological Studies (no. 100910001) as well as by the Ethics Committees of all participating institutions. All participants provided informed written consent in accordance with the Helsinki Declaration and other nationally valid regulations and guidelines. The JECS is conducted in accordance with the Helsinki Declaration and other nationally valid regulations and guidelines, and written informed consent was obtained from all participants or legal guardians of the participants under the age of 16 years. The present study included information of offspring up to 3 years of age, which were transcribed from medical records or provided by caregivers with parental consent. Authors did not have access to information that could identify individual participants during or after data collection.

Most of the questionnaires during pregnancy were distributed to women attending prenatal examinations, with some sent by post. Completed questionnaires were submitted during subsequent prenatal visits or mailed. When possible, respondents giving incomplete answers were interviewed face-to-face or by telephone for missing details. The first trimester and second/third questionnaire response rates were 98.5% and 97.2%, respectively [21]. Regarding the medical record transcriptions of mothers in early pregnancy and children at birth, the response rates were both 100% [21]. After the neonatal period, surveying has continued every 6 months via age-specific self-administered questionnaires mailed to mothers and caregivers. The completed questionnaires are returned to each Regional Center. If the questionnaire is not returned, reminders are sent by telephone, postcard, or text message as necessary by the Regional Center. As of September 25, 2022, the questionnaire response rates for 6-month-olds, 1-year-olds, and 3-year-olds are 94.1%, 91.4%, and 84.2%, respectively.

**Fig. 1:**
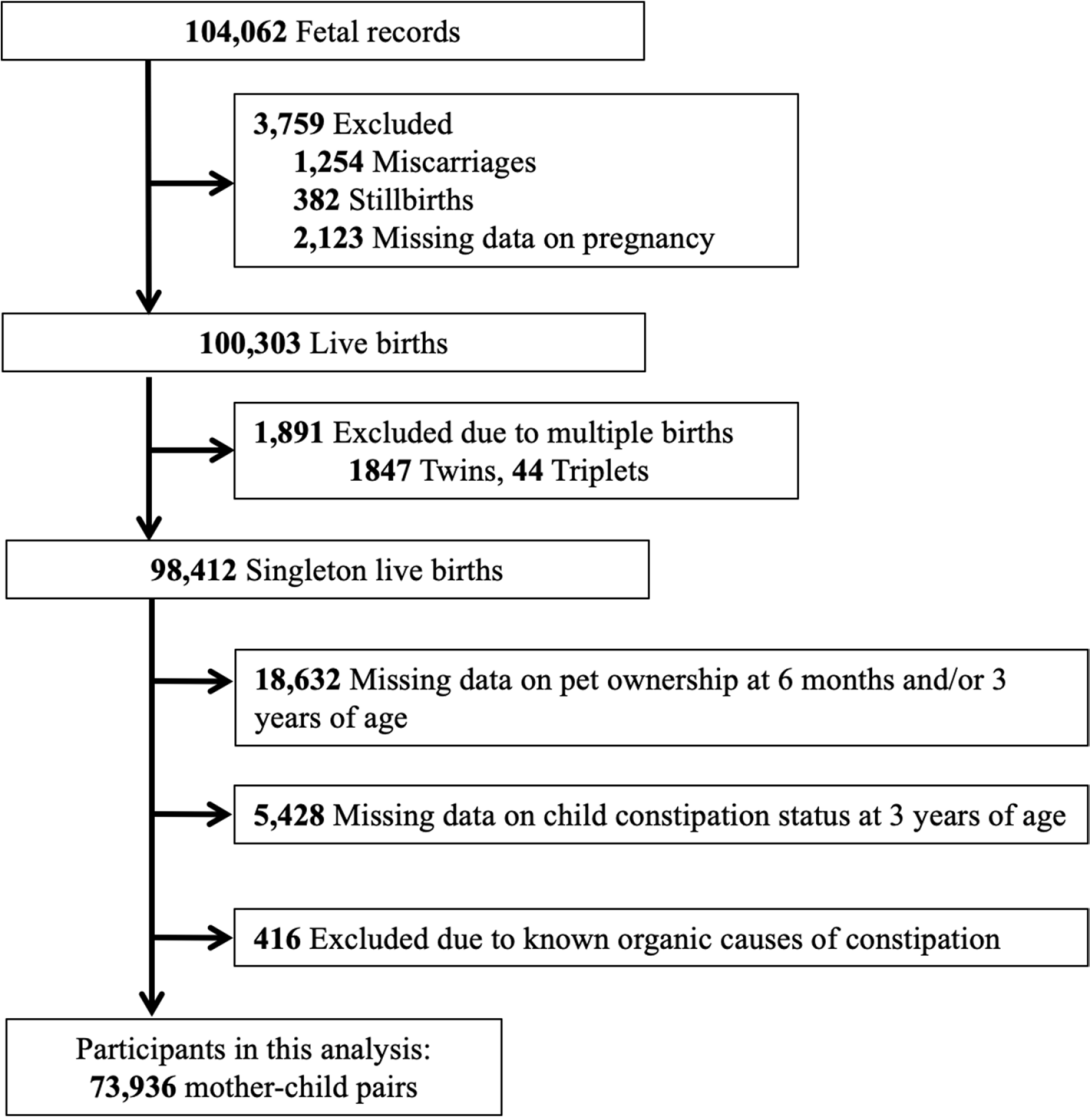
Case selection flowchart

The present study was based on the “jecs-ta-20190930” dataset released in October 2019. We accessed the data on December 18, 2019 and performed statistical analyzes between June 29, 2021 and February 1, 2023. The “jecs-ta-20190930” dataset contained information on 98,412 singleton live births up to 3 years of age (Fig. 1). We excluded 18,632 participants with insufficient or missing data on pet ownership at 6 months and/or 3 years of age and 5,428 participants with insufficient or missing data on the child’s constipation status. Participants reporting known organic causes of constipation, including Hirschsprung’s disease, spina bifida, thyroid gland insufficiency, and 21 trisomy as diagnosed by physicians, were also excluded, leaving 73,936 mother-toddler pairs for the analysis. The JECS sample size was confirmed to contain sufficient analytical power. For instance, to test a hypothesis concerning a disorder with a prevalence of 0.1% such as Down syndrome, a relative risk of 2.0, and an alpha error of 0.05 using a cohort in which the proportion of individuals with a high level of exposure to the chemical substance of interest is 25%, a sample of 67,503 participants would be required to provide a statistical power of 80% [24].

### Data collection

#### Exposure

Data on the child’s situation regarding dog and/or cat ownership were derived from the self-reported questionnaires at 6 months and 3 years of age. At 6 months of age, the question (translated from Japanese) was as follows: “Have you kept any pets in your house since your child started living at home (after birth and discharge from the hospital)? If yes, please indicate which one(s).” At 3 years of age, the question was as follows: “Do you keep pets at home? If yes, please indicate which one(s).” Participants who checked dog and/or cat were defined as dog and/or cat owners.We divided the dog and/or cat ownership period into the following 4 categories: non-ownership, infant-period ownership (until 6 months old only), current ownership (at 3 years old only), and long-term ownership (both until 6 months old and at 3 years old).

#### Outcomes

The primary outcome of interest was FC in 3-year-old toddlers according to the Rome III diagnostic criteria obtained from the self-reported questionnaires at 3 years of age [25, 26]. Rome III is an established set of diagnostic criteria to identify childhood functional gastrointestinal disorders, requiring a minimum of 2 of the following items lasting for at least1 month for a diagnosis of FC: 1) two or fewer defecations/week, 2) at least 1 episode/week of incontinence after acquiring toileting skills, 3) history of excessive stool retention, 4) history of hard or painful bowel movements, 5) large fecal mass in the rectum, and 6) history of large-diameter stools obstructing the toilet. We employed the Japanese version of Rome III in this investigation [27].

#### Covariates

The covariates in our models were selected a priori based on earlier published literature and biologic plausibility [4–6, 20, 28–30]. The following covariates for mothers and their partners were included: maternal age at delivery, pre-pregnancy body mass index (BMI), parity (primiparous or multiparous), cesarean birth (yes or no), maternal highest education age (< 16, 16 to < 19, or ≥ 19 years), marital status (married, single, divorced, or widowed), maternal gestational weight gain, annual household income (< 2 million, 2 million to < 4 million, 4 million to < 6 million, 6 million to < 8 million, or ≥ 8 million Japanese yen), and parental smoking status at 3 years of age (yes or no). For children, covariates included gender (male or female), birth weight, initial feeding habits during the first month of life (exclusively breastfeeding, partially breastfeeding, formula feeding, or other), started solid baby food at 6 months of age (yes or no), Kaup index at 3 years of age, wearing a diaper during sleep at 3 years of age (yes or no), attendance at preschool or childcare facility (daycare center/nursery) at 3 years of age (yes or no), and daily time playing outside during summer or winter from 2 years of age (almost none, < 1 h, 1–3 h, or ≥ 3 h). For all variables, the questionnaire content, respondent group and response period are summarized in S1 Table.

Pre-pregnancy BMI and Kaup index to evaluate body weight status for mothers and toddlers were calculated according to World Health Organization standards as body weight (kg)/height (m)^2^.

### Statistical analysis

The distribution normality of continuous variables was confirmed by the Kolmogorov–Smirnov test. Data are expressed as the mean ± standard deviation or the median (interquartile range) depending on whether they are normally distributed or not, respectively. The outcome of interest was FC defined as any toddler satisfying the Rome III diagnostic criteria as described by the mother. The exposure variables were non-ownership (reference), infant-period ownership, current ownership, and long-term ownership regarding dogs and/or cats. We adopted logistic regression models to calculate crude and adjusted odds ratios (ORs) and their 95% confidence intervals (CIs). The forced entry method was used to enter covariates into a multivariable analysis model. Spearman’s rank correlation coefficient was employed to check for multicollinearity of covariates. Hosmer–Lemeshow testing was used to assess the goodness-of-fit of the models. Model 1 was adjusted for demographic covariates, including maternal age at delivery, pre-pregnancy BMI, maternal highest level of education, annual household income, and marital status. Model 2 was adjusted for the covariates in model 1 in addition to perinatal covariates, including maternal gestational weight gain, parity, cesarean section at delivery, gender, birth weight, and breastfeeding initiation at 1 month of age. Model 3 was adjusted for the covariates in model 2 in addition to covariates in infancy, including started solid baby food at 6 months of age, parental smoking status at 3 years of age, Kaup index at 3 years of age, wearing a diaper during sleep at 3 years of age, attendance at preschool or childcare facility at 3 years of age, and daily time playing outside during summer after becoming 2 years of age. The JECS protocol prohibits covariate OR sharing to prevent double publications since other JECS studies use covariates as outcomes in parallel. Missing data on covariates were excluded in the logistic regression models. All statistical analyses were performed using SPSS statistical software version 28 (SPSS Inc., Chicago, Illinois). A *P*-value of < 0.05 was considered statistically significant.

## Results

A total of 73,936 mothers with singleton live births were available for analysis (Fig. 1). Table 1 summarizes the participants’ characteristics in terms of a history of dog and/or cat ownership. Among the participants, 57,264 (77.5%) had never owned a dog or cat before. We recorded 7,715 (10.4%) infant-period owners, 1,295 (1.8%) current owners, and 7,762 (10.5%) long-term owners. Significant differences were found among the groups regarding maternal age, pre-pregnancy BMI, gestational weight gain, annual household income, marital status, parental smoking habits at 3 years of age, parity, cesarean section, gestational age, initial feeding habits at 1 month of age, started solid baby food at 6 months of age, wearing a diaper at 3 years of age, attendance at preschool or childcare facility at 3 years of age, and duration playing outside (all *P* < 0.05).

**Table 1:** Characteristics of participants regarding history of dog/cat ownership (*N* = 73,936)

Variable	Dog and/or cat ownership			
	Non-ownership	Infant-period ownership (until 6 months of age)	Current ownership (at 3 years of age)	Long-term ownership (until 6 months and at 3 years of age)
	*N* = 57,264	*N* = 7,715	*N* = 1,295	*N* = 7,762
Maternal age at delivery (years)	32 (28, 35)	30 (26, 34)	30 (26, 34)	31 (28, 35)
Maternal age group, n (%)				
< 35 years	40,986 (71.6)	6,073 (78.7)	1,031 (79.6)	5,554 (72.5)
≥ 35 years	16,728 (28.4)	1,641 (21.3)	264 (20.4)	2,108 (27.5)
Pre-pregnancy BMI (kg/m^2^)	20.4 (19.1, 22.3)	20.5 (19.1, 22.5)	20.7 (19.1, 22.7)	20.6 (19.1, 22.7)
Pre-pregnancy BMI group, n (%)				
Underweight (BMI < 18.5)	9,338 (16.3)	1,221 (15.8)	204 (15.8)	1,200 (15.7)
Normal weight (BMI 18.5–24.9)	42,503 (74.3)	5,679 (73.6)	943 (72.9)	5,563 (72.7)
Overweight (BMI 25.0–29.9)	4,248 (7.4)	606 (7.9)	101 (7.8)	664 (8.7)
Obese (BMI ≥ 30.0)	1,144 (2.0)	207 (2.7)	46 (3.6)	223 (2.9)
Highest level of maternal education, n (%)				
< 16 years	1,749 (3.1)	381 (5.0)	88 (6.9)	471 (6.2)
16 to < 19 years	15,982 (28.2)	2,668 (35.0)	469 (36.9)	2,717 (35.9)
≥ 19 + years	39,008 (68.7)	4,570 (60.0)	713 (56.1)	4,379 (57.9)
Annual household income ^a^, n (%)				
< 2,000,000 JPY	2,181 (4.0)	478 (6.6)	107 (8.6)	430 (5.9)
2,000,000–3,999,999 JPY	14,911 (27.2)	2,395 (32.9)	430 (34.7)	2,273 (31.3)
4,000,000–5,999,999 JPY	19,729 (36.0)	2,464 (33.9)	385 (31.1)	2,468 (34.0)
6,000,000–7,999,999 JPY	10,496 (19.2)	1,123 (15.4)	194 (15.7)	1,205 (16.6)
≥ 8,000,000 JPY	128 (8.1)	817 (12.2)	122 (9.9)	890 (12.2)
Marital status, n (%)				
Married	55,033 (96.9)	7,140 (93.6)	1,208 (94.6)	7,168 (94.3)
Single	1,429 (2.5)	430 (5.6)	54 (4.2)	373 (4.9)
Divorced	333 (0.6)	58 (0.8)	15 (1.2)	56 (0.7)
Widowed	8 (0.0)	3 (0.0)	0 (0.0)	1 (0.0)
Body weight gain during pregnancy (kg)	10.1 (7.9, 12.4)	10.6 (8.2, 13.0)	10.7 (8.5, 13.0)	10.5 (8.1, 13.0)
Parity, n (%)				
Primiparous	22,231 (39.7)	3,783 (50.4)	433 (34.3)	3,510 (47.1)
Multiparous	33,697 (60.3)	3,727 (49.6)	828 (65.7)	3,938 (52.9)
Cesarean section at delivery, n (%)				
No	46,678 (81.9)	6,345 (82.6)	1,069 (82.9)	6,106 (80.0)
Yes	10,331 (18.1)	1,338 (17.4)	220 (17.1)	1,528 (20.0)
Male, n (%)	29,121 (50.9)	3,921 (50.8)	651 (50.3)	3,919 (51.1)
Gestational age (weeks)	39 (38, 40)	39 (38, 40)	39 (38, 40)	39 (38, 40)
Gestational age category, n (%)				
< 37 weeks	2,541 (4.4)	291 (3.8)	53 (4.1)	338 (4.4)
37–41 weeks	54,488 (95.3)	7,387 (96.0)	1,235 (95.5)	7,293 (95.3)
≥ 42 weeks	128 (0.2)	17 (0.2)	5 (0.4)	20 (0.3)
Birth weight (g)	3,030 (2,786, 3,282)	3,035 (2,786, 3,288)	3,064 (2,800, 3,300)	3,028 (2,786, 3,284)
Birth weight category, n (%)				
< 1,500 g	262 (0.5)	23 (0.3)	3 (0.2)	28 (0.4)
1,500–2,499 g	4,167 (7.3)	506 (6.6)	93 (7.2)	589 (7.7)
2,50–3,999 g	52,214 (91.4)	7,093 (92.2)	1,183 (91.6)	6,974 (91.2)
≥ 4000 g	485 (0.8)	67 (0.9)	12 (0.9)	57 (0.8)
Initial feeding habit at 1 month of age, n (%)				
Exclusively breastfeeding (reference)	31,652 (57.1)	3,899 (52.3)	671 (53.4)	3,841 (51.9)
Partially breastfeeding	21,971 (39.7)	3,287 (44.0)	522 (41.6)	3,214 (40.5)
Formula feeding	1,782 (3.2)	276 (3.7)	63 (5.0)	352 (4.8)
Started solid baby food at 6 months of age, n (%)				
No	41,399 (72.4)	5,982 (77.7)	1,007 (78.2)	5,900 (77.2)
Yes	15,759 (27.6)	1,714 (22.3)	281 (21.8)	1,744 (22.8)
Kaup index at 3 years of age (kg/m^2^)	15.9 (15.2, 16.8)	16.0 (15.2, 16.8)	16.0 (15.2, 16.8)	15.9 (15.2, 16.8)
Maternal smoking at 3 years of age, n (%)				
No	52,113 (91.7)	6,662 (87.4)	1,059 (82.9)	6,510 (85.9)
Yes	4,713 (8.3)	960 (12.6)	219 (17.1)	1,068 (6.4)
Partner smoking at 3 years of age, n (%)				
No	36,855 (66.0)	4,240 (57.0)	636 (51.2)	4,104 (55.4)
Yes	18,988 (34.0)	3,197 (43.0)	605 (48.8)	3,309 (44.6)
Wearing a diaper during sleep at 3 years of age, n (%)				
No	8,052 (14.1)	1,057 (13.7)	192 (14.8)	970 (12.7)
Yes	4,9136 (85.9)	6,647 (86.3)	1,102 (85.2)	6,682 (87.3)
Attendance at preschool or childcare facility (daycare center/nursery) at 3 years of age, n (%)				
No	20,400 (36.8)	2,524 (33.8)	408 (32.8)	2,696 (36.2)
Yes	35,106 (63.2)	4,949 (66.2)	835 (67.2)	4,743 (63.8)
Daily hours playing outside during summer after becoming 2 years of age, n (%)				
Almost none	1,141 (2.0)	122 (1.6)	21 (1.7)	183 (2.4)
< 1 h	13,566 (24.2)	1,690 (22.5)	294 (23.3)	1,704 (22.8)
1–3 h	37,202 (66.4)	5,040 (67.2)	821 (65.2)	4,932 (66.0)
≥ 3 h	4,089 (7.3)	653 (8.7)	124 (9.8)	655 (8.8)
Daily hours playing outside during winter after becoming 2 years of age, n (%)				
Almost none	5,756 (10.4)	776 (10.5)	115 (9.3)	780 (10.6)
< 1 h	24,667 (44.6)	3,351 (45.2)	549 (44.3)	3,216 (43.6)
1–3 h	23,317 (42.1)	3,074 (41.4)	525 (42.3)	3,139 (42.5)
≥ 3 h	1,624 (2.9)	217 (2.9)	51 (4.1)	243 (3.3)

Continuous variables are expressed as the median (interquartile range)

Data were missing on maternal age (*n* = 1), pre-pregnancy BMI (*n* = 46), maternal education level (*n* = 741), household income (*n* = 3,350), marital status (*n* = 627), body weight gain during pregnancy (*n* = 1,703), parity (*n* = 1,789), mode of delivery (*n* = 321), birth weight (*n* = 180), gestational age (*n* = 140), breastfeeding initiation at 1 month of age (*n* = 2,406), started solid baby food at 6 months of age (*n* = 150), Kaup index at 3 years of age (*n* = 140), maternal smoking habit (*n* = 632), partner smoking habit (*n* = 2,002), wearing a diaper during sleep at 3 years of age (*n* = 98), attendance at preschool or childcare facility (daycare center/nursery) at 3 years of age (*n* = 2,275), Daily hours playing outside during summer after becoming 2 years of age (*n* = 1,699), and daily hours playing outside during winter after becoming 2 years of age (*n* = 2,536)

*BMI* Body mass index, *JPY* Japanese yen

^a^ The average (median) annual Japanese household income in 2018 was 5,523,000 JPY (4,370,000 JPY). The currency exchange rates on January 25, 2023, were: 1 USD = 130 JPY and 1 EUR = 142 JPY

A total of 8,459 toddlers (11.6%) met the Rome III criteria at 3 years of age and were included in logistic regression analysis on the association between a history of dog and/or cat ownership and FC at 3 years of age (Table 2). Univariate analysis showed that infant-period ownership and long-term ownership significantly increased the risk of developing FC as compared with non-ownership (crude OR [95% CI] 1.15 [1.07 − 1.24], 1.12 [1.04–1.20], respectively). Additionally, infant-period ownership remained significantly associated with the risk of developing FC at 3 years of age after adjusting for covariates (adjusted OR [95% CI] 1.13 [1.05–1.22] in model 1, 1.11 [1.02–1.20] in model 2, and 1.09 [1.01–1.19] in model 3, respectively, based on non-ownership).

**Table 2:** Associations between dog and/or cat ownership and functional constipation

Variable	Functional constipation		Univariate	Multivariate		
	No, n (%)	Yes, n (%)	Crude OR (95% CI)	Model 1	Model 2	Model 3
	65,325 (88.4)	8,459 (11.6)		Adjusted OR (95% CI)	Adjusted OR (95% CI)	Adjusted OR (95% CI)
Dog and/or cat ownership						
Non-ownership	50,745 (88.6)	6,519 (11.4)	1.00	1.00	1.00	1.00
Infant-period ownership (until 6 months of age)	6,721 (87.1)	994 (12.9)	**1.15 (1.07–1.24)**	**1.13 (1.05–1.22)**	**1.11 (1.02–1.20)**	**1.09 (1.01–1.19)**
Current ownership (at 3 years of age)	1,157 (89.3)	138 (10.7)	0.93 (0.78–1.11)	0.89 (0.74–1.07)	0.92 (0.76–1.12)	0.80 (0.80–1.22)
Long-term ownership (until 6 months and at 3 years of age)	6,702 (87.5)	960 (12.5)	**1.12 (1.04–1.20)**	**1.08 (1.00–1.16)**	1.07 (0.99–1.15)	1.01 (0.99–1.02)

Model 1 was adjusted for demographic covariates, including maternal age at delivery, pre-pregnancy body mass index, maternal highest level of education, annual household income at 3 years of age, and marital status

Model 2 was adjusted for the covariates in model 1 in addition to perinatal covariates, including maternal gestational weight gain, parity, caesarian section at delivery, gender, birth weight, gender, and breastfeeding initiation at 1 month of age

Model 3 was adjusted for the covariates in model 2 in addition to covariates in infancy, including started solid baby food at 6 months of age, parental smoking status at 3 years of age, Kaup index at 3 years of age, wearing a diaper during sleep at 3 years of age, attendance at preschool or childcare facility (daycare center/nursery) at 3 years of age, and daily time playing outside during summer after becoming 2 years of age

*OR* Odds ratio, *CI* Confidence interval

## Discussion

This study describes the first large-scale nationwide birth cohort study in Japan examining the relationship of family dog and/or cat ownership status early in life with FC at 3 years of age. Our results indicate that infant-period dog and/or cat ownership prior to 6 months of age may significantly increase the risk of FC at 3 years of age.

Childhood constipation is reportedly more frequent during the weaning period, toilet training in toddlers, and in school-childhood when children start school but avoid defecation there [31]. FC onset is most likely while toilet training around 3 years of age [31]. Dietary habits, fluid intake, exercise, socioeconomic variables, and psychological factors all affect defecation pattern in young children [3–5]. Nakamura et al. also revealed that toddlers born by cesarean delivery had a significantly higher risk of suffering FC than those born by vaginal delivery [32], possibly by a mechanism of variability in intestinal microbiota composition [33, 34]. In a prospective investigation, the gut microbiota of children with FC diagnosed by Rome III criteria were clearly discriminated from those of healthy controls, and so alterations in intestinal microbiota might factor prominently in pediatric FC occurrence [20, 35, 36].

Mutually beneficial relationships between humans and animals have been widely reported as human-animal interactions. The psychological, physiological, social, and communicative benefits of interaction with animals have all been established, with the potential for animal-assisted therapy in a wide range of fields being reported as well [7–13]. In children, exposure to pets during the first year of life has been found to reduce the risk of allergic rhinitis and asthma at school age [37]. Azad et al. demonstrated that early life exposure to household pets might influence the overall diversity and composition of infant gut microbiota, with potential implications in the development of atopy [16]. Heyworth et al. reported that having a dog or cat was associated with a reduced risk of gastroenteritis in young children and postulated the mechanism of acquired immunity from low-level chronic exposure to microorganisms carried by the animal [13]. Other studies have described the effects of household pet ownership on gut microbial composition versus non-pet controls [14–19]. It has been suggested that the diversity of the gut microbiota, particularly within Proteobacteria, may be reduced, as domestic pet-mediated exposure to the natural environment leads to overgrowth of certain microbes [15, 19]. An imbalance in intestinal flora is thought to be an etiological factor contributing to FC in children. It is also possible that a decrease in the diversity of gut microbiota in pet owners is related to the development of FC.

We observed that infant-period dog and/or cat ownership prior to 6 months of age might be associated with a higher risk of FC at 3 years of age. Tun et al. revealed changes in the taxon abundance of gut microbiota in infants exposed to furry pets during pregnancy or the postnatal period [15]. At the minimum, our findings suggested that early gut microbiome disturbances influenced by attachment with a household dog and/or cat could increase the risk of developing FC later. Long-term pet ownership, which included infancy and current ownership, showed a significant association with FC development after controlling for demographic variables. However, when adjusting for covariates from the perinatal period onwards, this apparent tendency lost statistical significance. Further research is needed on the effect of continued long-term dog and/or cat ownership on the intestinal environment, including such confounding factors after infancy as information on dietary habits, fluid intake, and physical activity.

This study had several limitations. First, the data as measured by Rome III were collected from mother and caregiver self-reported questionnaires and therefore considered subjective. Additionally, data were not available on whether FC was diagnosed or treated by doctors, creating a possibility of recall bias or erroneous interpretation. Second, as the findings on FC were evaluated at 3 years of age, children with FC onset and improvement earlier or later than that age were not considered. Third, the large attrition rate of participants who lacked information on having household pets as well as those not completing Rome III may have constituted selection bias; we cannot conclusively rule out the possibility of under-reporting the incidence of FC. Fourth, there may be unadjusted confounding factors, such as a history of antibiotic, probiotic, or prebiotic use by the mother in the gestational period or child, which affected gut microbiota profiles. Fifth, among the participants of this study, 87% of those who answered that they had any pets had dogs and/or cats, therefore, we did not consider the impact of other minority domestic pets (rodents, birds, reptiles and insects) and livestock on developing FC in children. Lastly and possibly most importantly, information on dietary habits that play a considerable role in the development of constipation, including fruit, vegetable, water, and fast food consumption, in addition to the genetic background of constipation, were unavailable and could not be included as covariates.

## Conclusions

In conclusion, this is the first study employing a large dataset from a Japanese nationwide birth cohort study to analyze the impact of dog and/or cat ownership status early in life on the presence of FC at 3 years of age. After controlling for potential confounders, living with dog and/or cat during early infancy was identified to possibly increase the risk of FC. Such findings may constitute clinical evidence on the relationship and interaction between children and their household pets. Further research is needed to determine how long-term pet ownership including early infancy affects the subsequent intestinal environment.

### Supplementary Information

**Additional file 1: S1 Table.** Questionnaire Content.

## Data Availability

Data are unsuitable for public deposition due to ethical restrictions and legal framework of Japan. It is prohibited by the Act on the Protection of Personal Information (Act No. 57 of 30 May 2003, amendment on 9 September 2015) to publicly deposit the data containing personal information. Ethical Guidelines for Medical and Health Research Involving Human Subjects enforced by the Japan Ministry of Education, Culture, Sports, Science and Technology and the Ministry of Health, Labour and Welfare also restricts the open sharing of the epidemiologic data. All inquiries about access to data should be sent to: jecs-en@nies.go.jp. The person responsible for handling enquiries sent to this e-mail address is Dr Shoji F. Nakayama, JECS Programme Office, National Institute for Environmental Studies.

## Abbreviations

BMI: Body mass index
CI: Confidence interval
FC: Functional constipation
JECS: The Japan Environment and Children’s Study
OR: Odds ratio

## References

[1] Rahhal R, Aliye UC, Kleinman ER, Sanderson RI, Goulet O (2008). Functional constipation. Walker’s pediatric gastrointestinal disease.

[2] Koppen IJN, Vriesman MH, Saps M (2018). Prevalence of functional defecation disorders in children: a systematic review ajd meta-analysis. J Pediatr.

[3] Park M, Bang YG, Cho KY (2016). Risk factors for functional constipation in young children attending daycare centers. J Korean Med Sci.

[4] Rajindrajith S, Devanarayana NM, Benninga MA (2017). Constipation and constipation-predominant irritable bowel syndrome: a comparative study using Rome III criteria. J Pediatr Gastroenterol Nutr.

[5] Inan M, Aydiner CY, Tokuc B, Aksu B, Ayvaz S, Ayhan S (2007). Factors associated with childhood constipation. J Paediatr Child Health.

[6] American Veterinary Medical Association (2018). AVMA pet ownership and demographics sourcebook.

[7] Taniguchi Y, Seino S, Nishi M, Tomine Y, Tanaka I, Yokoyama Y (2018). Physical, social, and psychological characteristics of community-dwelling elderly Japanese dog and cat owners. PLoS One.

[8] Mubanga M, Byberg L, Nowak C, Egenvall A, Magnusson PK, Ingelsson E, Fall T (2017). Dog ownership and the risk of cardiovascular disease and death - a nationwide cohort study. Sci Rep.

[9] Smith B (2012). The ‘pet effect’--health related aspects of companion animal ownership. Aust Fam Physician.

[10] Christian H, Trapp G, Lauritsen C, Wright K, Giles-Corti B (2013). Understanding the relationship between dog ownership and children’s physical activity and sedentary behavior. Pediatr Obes.

[11] Minatoya M, Ikeda-Araki A, Miyashita C, Itoh S, Kobayashi S, Yamazaki K (2021). Association between early life child development and family dog ownership: a prospective birth cohort study of the Japan Environment and Children’s Study. Int J Environ Res Public Health.

[12] Bergroth E, Remes S, Pekkanen J, Kauppila T, Büchele G, Keski-Nisula L (2012). Respiratory tract illnesses during the first year of life: effect of dog and cat contacts. Pediatrics.

[13] Heyworth JS, Cutt H, Glonek G (2006). Does dog or cat ownership lead to increased gastroenteritis in young children in South Australia?. Epidemiol Infect.

[14] Du G, Huang H, Zhu Q, Ying L (2021). Effects of cat ownership on the gut microbiota of owners. PLoS One.

[15] Tun HM, Konya T, Takaro TK, Brook JR, Chari R, Field CJ (2017). Exposure to household furry pets influences the gut microbiota of infant at 3–4 months following various birth scenarios. Microbiome.

[16] Azad MB, Konya T, Maughan H, Guttman DS, Field CJ, Sears MR (2013). Infant gut microbiota and the hygiene hypothesis of allergic Disease: impact of household pets and siblings on microbiota composition and diversity. Allergy Asthma Clin Immunol.

[17] Kates AE, Jarrett O, Skarlupka JH, Sethi A, Duster M, Watson L (2020). Household pet ownership and the microbial diversity of the human gut microbiota. Front Cell Infect Microbiol.

[18] Lehtimäki J, Sinkko H, Hielm-Björkman A, Laatikainen T, Ruokolainen L, Lohi H (2020). Simultaneous allergic traits in dogs and their owners are associated with living environment, lifestyle and microbial exposures. Sci Rep.

[19] Nielsen CC, Gascon M, Osornio-Vargas AR, Shier C, Guttman DS, Becker AB (2020). Natural environments in the urban context and gut microbiota in infants. Environ Int.

[20] de Meij TG, de Groot EF, Eck A, Budding AE, Kneepkens CM, Benninga MA (2016). Characterization of Microbiota in children with chronic functional constipation. PLoS One.

[21] Michikawa T, Nitta H, Nakayama SF, Yamazaki S, Isobe T, Tamura K (2018). Baseline profile of participants in the Japan Environment and Children’s Study (JECS). J Epidemiol.

[22] Ishitsuka K, Nakayama SF, Kishi R, Mori C, Yamagata Z, Ohya Y (2017). Japan Environment and Children’s Study: backgrounds, activities, and future directions in global perspectives. Environ Health Prev Med.

[23] Kawamoto T, Nitta H, Murata K, Toda E, Tsukamoto N, Hasegawa M (2014). Rationale and study design of the Japan Environment and Children’s Study (JECS). BMC Public Health.

[24] Japan Environment and Children’s Study (JECS) study protocol (ver.1.4). 2016. Available from: http://www.env.go.jp/chemi/ceh/en/about/advanced/material/jecs-study_protocol_14_en.pdf . Accessed 20 June 2023.

[25] Drossman DA, Corazziari E, Delvaux M, Spiller RC, Talley NJ, Thompson WG (2006). Rome III: the Functional gastrointestinal disorders.

[26] Hyman PE, Milla PJ, Benninga MA, Davidson GP, Fleisher DF, Taminiau J (2006). Childhood functional gastrointestinal disorders: neonate/toddler. Gastroenterology.

[27] Fukudo S, Hongo M, Matsueda K (2008). Rome III: The functional gastrointestinal disorders.

[28] Werth BL, Christopher SA (2021). Potential risk factors for constipation in the community. World J Gastroenterol.

[29] Yoshida T, Matsumura K, Tsuchida A, Hamazaki K, Inadera H, Japan Environment and Children’s Study Group (2018). Association between cesarean section and constipation in infants: the Japan Environment and Children’s Study (JECS). BMC Res Notes.

[30] Steutel NF, Zeevenhooven J, Scarpato E, Vandenplas Y, Tabbers MM, Staiano A (2020). Prevalence of functional gastrointestinal disorders in European infants and toddlers. J Pediatr.

[31] Di Lorenzo C (2001). Pediatric anorectal disorders. Gastroenterol Clin North Am.

[32] Nakamura M, Matsumura K, Ohnuma Y (2021). Association of cesarean birth with prevalence of functional constipation in toddlers at 3 years of age: results from the Japan Environment and Children’s Study (JECS). BMC Pediatr.

[33] Azad MB, Konya T, Maughan H (2013). Gut microbiota of healthy Canadian infants: profiles by mode of delivery and infant diet at 4 months. CMAJ.

[34] Salminen S, Gibson GR, McCartney AL, Isolauri E (2004). Influence of mode of delivery on gut microbiota composition in seven year old children. Gut.

[35] Avelar Rodriguez D, Popov J, Ratcliffe EM, Toro Monjaraz EM (2021). Functional constipation and the gut microbiome in children: preclinical and clinical evidence. Front Pediatr.

[36] Jomehzadeh N, Javaherizadeh H, Amin M, Rashno M, Teimoori A (2020). Quantification of intestinal Lactobacillus species in children with functional constipation by quantitative real-time PCR. Clin Exp Gastroenterol.

[37] Ownby DR, Johnson CC, Peterson EL (2002). Exposure to dogs and cats in the first year of life and risk of allergic sensitization at 6 to 7 years of age. JAMA.

