# Supplementary material for: Impact of dog and/or cat ownership on functional constipation at 3 years of age: the Japan Environment and Children’s study
Source: BMC Pediatr. 2023 Nov 23;23:595. doi: 10.1186/s12887-023-04412-4 (PMC10666348; doi:10.1186/s12887-023-04412-4)
Supplement: Supplementary file 1 — Additional file 1: S1 Table. Questionnaire Content. [file 12887_2023_4412_MOESM1_ESM.docx]

**Supplementary material**

**S1 Table.** Questionnaire Content

| **Questionnaire** | **Respondents** | **Response period** | **Question** | **Answer** |
| --- | --- | --- | --- | --- |
| MT1 | Mother | In the 1st trimester | Please tell us about your current marital status. (Check only one) | 1 = Married (including common-low marriage), 2 = Single (Never married) , 3 = Divorced, 4 = Widowed |
| DrT1 | Doctor, midwife, or nurse | In the 1st trimester | Mother's height |  |
|  |  |  | Mother's weight before pregnancy |  |
|  |  |  | Number of previous pregnancies |  |
|  |  |  | Number of previous deliveries |  |
| MT2 | Mother | In the 2nd/third trimester | Highest level of education | 1 = Junior high school, 2 = High school, 3 = Technical junior college, 4 = Technical/vocational college, 5 = Associate degree, 6 = Bachelor’s degree, 7 = Graduate degree (Master’s/Doctor’s) |
| Dr0m | Doctor, midwife, or nurse | At birth | Age of mother |  |
|  |  |  | Pregnancy outcome | 1 = single birth, 2 = multiple birth |
|  |  |  | Number of babies born together |  |
|  |  |  | Weeks and days of pregnancy at the time of delivery: ___________weeks |  |
|  |  |  | Weeks and days of pregnancy at the time of delivery: ___________days |  |
|  |  |  | Whether the baby was alive | 1 = Live birth, 2 = Stillbirth |
|  |  |  | Sex | 1 = Male, 2 = Female, 3 = Indeterminate |
|  |  |  | Apgar score at 1 minute after birth |  |
|  |  |  | Apgar score at 5 minutes after birth |  |
|  |  |  | Mode of delivery | 1 = Spontaneous delivery, 2 = Induced delivery, 3 = Vacuum extraction, 4 = Forceps delivery, 5 = Planned Cesarean delivery/Emergent Cesarean delivery |
|  |  |  | Weight at birth(g) |  |
|  |  |  | Height at birth(cm) |  |
|  |  |  | Mother's weight before pregnancy: ____________kg |  |
|  |  |  | Date of weight measurement |  |
|  |  |  | Pregnancy week ___________ week |  |
|  |  |  | Mother's weight immediately before delivery: ________kg |  |
|  |  |  | Date (when the weight was measured) |  |
|  |  |  | Week of pregnancy (when the weight was measured) |  |
|  |  |  | Physical anomalies | 1 = No, 2 = Yes |
|  |  |  | Down's syndrome | 0 = No, 1 = Yes |
|  |  |  | Imperforate anus (anorectal anomaly) | 0 = No, 1 = Yes |
|  |  |  | Myelomeningocele (spina bifida) | 0 = No, 1 = Yes |
| Dr1m | Doctor, midwife, or nurse | When the children was 1 month of age | Methods of feeding: Breastfeeding | 0 = No, 1 = Yes |
|  |  |  | Mixed feeding | 0 = No, 1 = Yes |
|  |  |  | Infant formula | 0 = No, 1 = Yes |
|  |  |  | Physical anomalies | 1 = No, 2 = Yes |
|  |  |  | Down's syndrome | 0 = No, 1 = Yes |
|  |  |  | Imperforate anus (anorectal anomaly) | 0 = No, 1 = Yes |
|  |  |  | Myelomeningocele (spina bifida) | 0 = No, 1 = Yes |
|  |  |  | Congenital hypothyroidism (cretinism) | 0 = No, 1 = Yes |
| C6m | Caregivers | When the children was 6 months of age | Started solids (including fruit juice or rice gruel) | 1 = Yes, 2 = No |
|  |  |  | Gastrointestinal disease :  (e.g., Hypertrophic pyloric stenosis, Biliary atresia, Hirschsprung's disease, Intussusception) | 0 = No, 1 = Yes |
|  |  |  | Having pet(s) at home (either currently or in the past) | 1 = No, 2 = Yes |
|  |  |  | Dog(s) (kept inside the house) | 0 = No, 1 = Yes |
|  |  |  | Dog(s) (kept outside the house) | 0 = No, 1 = Yes |
|  |  |  | cat(s) | 0 = No, 1 = Yes |
| C1Y | Caregivers | When the children was 1 year of age | Spinal cord disorder (e.g., spinal bifida) | 0 = No, 1 = Yes |
|  |  |  | Name of the spinal cord disorder |  |
|  |  |  | Gastrointestinal disorder or chest disorder (e.g., kinguinal hernia, esophageal atresia, biliary atresia, Hirschsprung disease, anal atresia) | 0 = No, 1 = Yes |
|  |  |  | Name of the gastrointestinal disorder or chest disorder |  |
|  |  |  | Endocrine disorder (e.g., congenital adrenocortical hyperplasia, hypothyroidism, cretinism) | 0 = No, 1 = Yes |
|  |  |  | Name of the endocrine disorder |  |
|  |  |  | Chromosomal anomaly (e.g., Down’s syndrome, trisomy 18) | 0 = No, 1 = Yes |
|  |  |  | Name of the chromosomal anomaly |  |
|  |  |  | Cerebral palsy | 0 = No, 1 = Yes |
| C2Y | Caregivers | When the children was 2 years of age | Spinal cord disorder (e.g., spinal bifida) | 0 = No, 1 = Yes |
|  |  |  | Name of the spinal cord disorder |  |
|  |  |  | Gastrointestinal disorder or chest disorder (e.g., kinguinal hernia, esophageal atresia, biliary atresia, Hirschsprung disease, anal atresia) | 0 = No, 1 = Yes |
|  |  |  | Name of the gastrointestinal disorder or chest disorder |  |
|  |  |  | Endocrine disorder (e.g., congenital adrenocortical hyperplasia, hypothyroidism, cretinism) | 0 = No, 1 = Yes |
|  |  |  | Name of the endocrine disorder |  |
|  |  |  | Chromosomal anomaly (e.g., Down’s syndrome, trisomy 18) | 0 = No, 1 = Yes |
|  |  |  | Name of the chromosomal anomaly |  |
| C3Y | Caregivers | When the children was 3 years of age | Current height: _____ cm |  |
|  |  |  | Current weight: ______kg |  |
|  |  |  | Endocrine and metabolic disorder (e.g., growth hormone disorder, thyroid disorder, diabetes) | 0 = No, 1 = Yes |
|  |  |  | Neurological disorder diagnosed after age 2: Celebral palsy | 0 = No, 1 = Yes |
|  |  |  | Does your child wear a diaper at night? | 1 = Yes, 2 = No |
|  |  |  | Number of hours of playing outside during daytime (9:00-17:00) per day after becoming age 2, in the summer (between May and September) | 1 = Almost none, 2 = less than 1 hour, 3 = 1-3 hours, 4 = more than 3 hours, 5 = I don't know |
|  |  |  | Number of hours of playing outside during daytime (9:00-17:00) per day after becoming age 2, in the winter (between November and February) | 1 = Almost none, 2 = less than 1 hour, 3 = 1-3 hours, 4 = more than 3 hours, 5 = I don't know |
|  |  |  | Attendance at a preshool or a childcare facility (daycare center or nursery) | 1 = Yes, 2 = No |
|  |  |  | I (mother) currently smoke | 1 = Yes, 2 = No |
|  |  |  | Are there other individuals within the household who smoke? If so, how many cigarettes are smoked inside the house per day. (If there is more than one individual who smokes, record an estimate amount of cigarettes smoked within the house per day) | 1 = Yes, 2 = No |
|  |  |  | Annual household income | 1 = less than 2 million yen, 2 = 2 million yen to less than 4 million yen, 3 = 4 million yen to less than 6 million yen, 4 = 6 million yen to less than 8 million yen, 5 = 8 million yen to less than 10 million yen, 6 = 10 million yen to less than 12 million yen, 7 = 12 million yen to less than 15 million yen, 8 = 15 million yen to less than 20 million yen, 9 = 20 million yen and over |
|  |  |  | a. Defecates (poos) less than twice a week | 1 = Yes, 2 = No |
|  |  |  | b. Experiences bowel incontinencies more than once a week after growing out of diapers | 1 = Yes, 2 = No |
|  |  |  | c. Holds in poo sometimes | 1 = Yes, 2 = No |
|  |  |  | d. Passes hard stools or experiences pain during defecation | 1 = Yes, 2 = No |
|  |  |  | e. Experiences intestinal blockage from stools | 1 = Yes, 2 = No |
|  |  |  | f. Passes stools large enough to clog the toilet | 1 = Yes, 2 = No |
|  |  |  | Having pet(s) inside the house | 1 = Yes, 2 = No |
|  |  |  | Pet(s) at home: Dog | 0 = No, 1 = Yes |
|  |  |  | Cat | 0 = No, 1 = Yes |
